# MECHANISMS IN ENDOCRINOLOGY: Human brown adipose tissue as a therapeutic target: warming up or cooling down?

**DOI:** 10.1530/EJE-20-1439

**Published:** 2021-03-10

**Authors:** Ben T McNeill, Karla J Suchacki, Roland H Stimson

**Affiliations:** 1 University/BHF Centre for Cardiovascular Science, University of Edinburgh, Queen’s Medical Research Institute, Edinburgh, UK

## Abstract

Excessive accumulation of white adipose tissue leads to obesity and its associated metabolic health consequences such as type 2 diabetes and cardiovascular disease. Several approaches to treat or prevent obesity including public health interventions, surgical weight loss, and pharmacological approaches to reduce caloric intake have failed to substantially modify the increasing prevalence of obesity. The (re-)discovery of active brown adipose tissue (BAT) in adult humans approximately 15 years ago led to a resurgence in research into whether BAT activation could be a novel therapy for the treatment of obesity. Upon cold stimulus, BAT activates and generates heat to maintain body temperature, thus increasing energy expenditure. Activation of BAT may provide a unique opportunity to increase energy expenditure without the need for exercise. However, much of the underlying mechanisms surrounding BAT activation are still being elucidated and the effectiveness of BAT as a therapeutic target has not been realised. Research is ongoing to determine how best to expand BAT mass and activate existing BAT; approaches include cold exposure, pharmacological stimulation using sympathomimetics, browning agents that induce formation of thermogenic beige adipocytes in white adipose depots, and the identification of factors secreted by BAT with therapeutic potential. In this review, we discuss the caloric capacity and other metabolic benefits from BAT activation in humans and the role of metabolic tissues such as skeletal muscle in increasing energy expenditure. We discuss the potential of current approaches and the challenges of BAT activation as a novel strategy to treat obesity and metabolic disorders.

## Invited Author’s profile


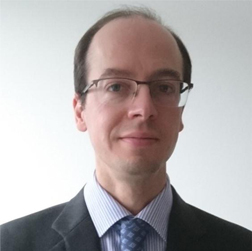


**Roland H Stimson** is Professor of Endocrinology and a Scottish Senior Clinical Fellow at the University of Edinburgh and an honorary consultant physician at the Royal Infirmary of Edinburgh, UK. His research background is in human experimental medicine and integrative physiology. Prof Stimson’s overarching research interest is in obesity and its associated metabolic disease. His laboratory’s current focus is on investigating the role of human brown and white adipose tissue and skeletal muscle in the regulation of energy balance with a goal to identify novel pathways amenable to therapeutic manipulation to develop new treatments for obesity and type 2 diabetes mellitus.

## Introduction

**Figure 1:**
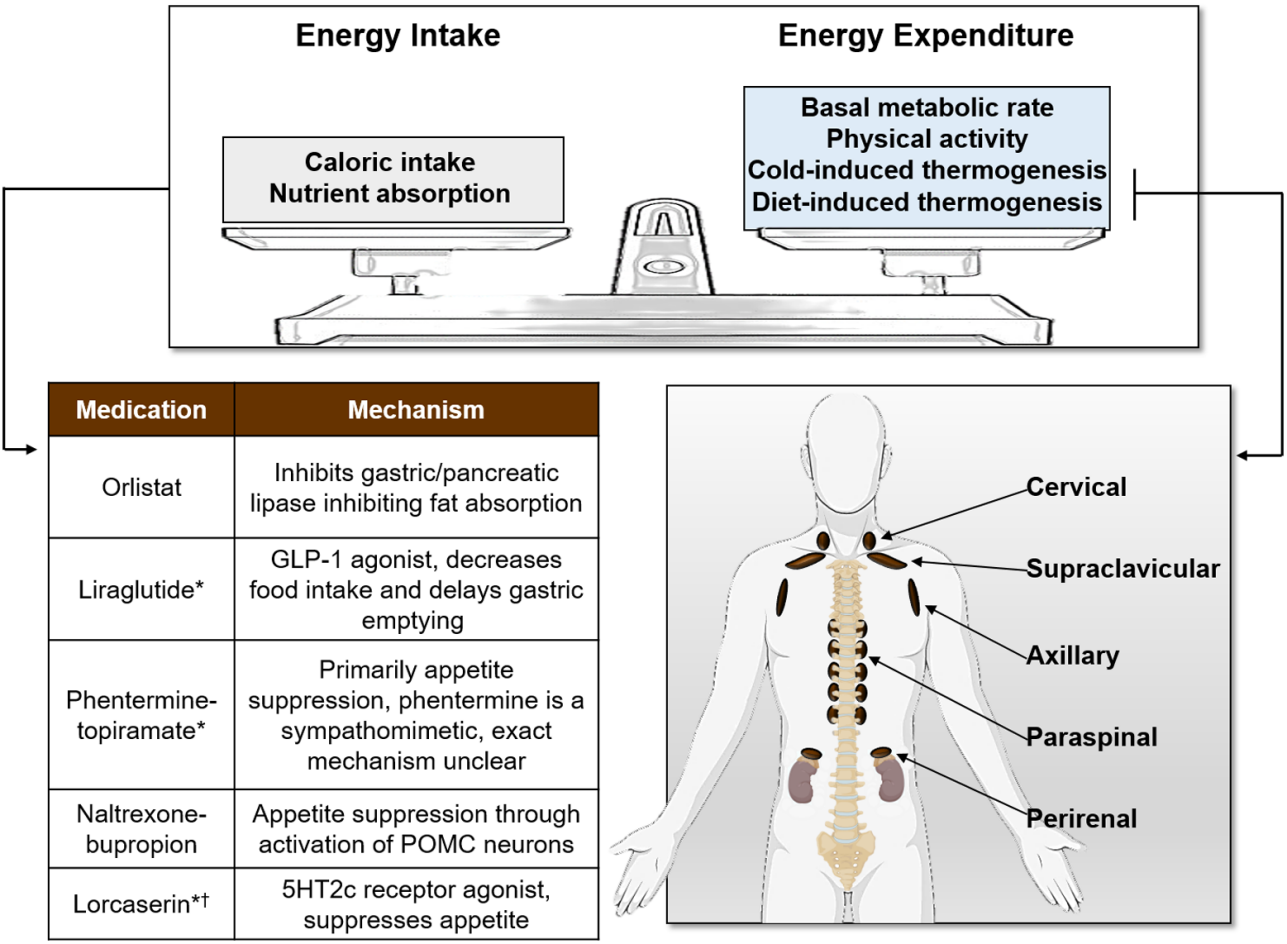
The energy balance equation and current pharmacotherapy to achieve weight loss. Energy balance is governed by the relationship between energy input (calories consumed) and energy output (energy expended). Obesity results from a chronic imbalance of energy intake exceeding energy expenditure with storage of this excess energy as triglycerides mainly in white adipose tissue. All licensed anti-obesity medications primarily cause weight loss by reducing appetite/energy intake (*indicates licensed to treat obesity in US only, ^†^indicates currently withdrawn). Basal metabolic rate, physical activity, diet-induced thermogenesis (DIT) and cold-induced thermogenesis (CIT) all contribute to total energy expenditure. Brown adipose tissue (BAT) is located in adult humans primarily in the cervical, supraclavicular, axillary, paravertebral and peri-renal regions. BAT activation is a key component of both CIT and DIT and is an attractive target to increase energy expenditure to treat obesity. 5HT, 5-hydroxytryptamine; GLP-1, glucagon-like peptide 1; POMC, pro-opiomelanocortin.

The prevalence of obesity has increased dramatically over the past 40 years, with over a quarter of adults in the UK now classed as obese (1). During the same period of time, global obesity has increased more than three-fold and this is likely to continue (2, 3). Obesity (defined as a BMI ≥30 kg/m^2^) occurs when energy intake chronically exceeds energy expenditure (EE) with deposition of this excess energy primarily as triglycerides in white adipose tissue (WAT). Obesity substantially increases the risk of developing other diseases such as type 2 diabetes mellitus (T2DM), hypertension, dyslipidaemia and cardiovascular disease and decreases both life expectancy and quality (4, 5). Public health interventions to promote weight loss through dietary caloric restriction and increased physical activity have failed to curb the rise in obesity due to lack of adherence (5). Pharmacological anti-obesity agents have focused on decreasing energy intake/appetite but have had limited success, in part due to serious adverse side effects leading to their withdrawal (most recently Lorcaserin) (6). Orlistat (a pancreatic lipase inhibitor) and naltrexone-bupropion are the only licensed medications in the UK for obesity and substantial side effects limit patient compliance; other agents are licensed in the USA but are not approved in Europe (Fig. 1) (7). Bariatric surgery can successfully treat obesity (8); however, these procedures are invasive, can cause significant complications and are not suitable for everyone.

There has been less effort on the development of pharmacotherapy to specifically increase EE (energy balance equation; Fig. 1). However, the use of ^18^F-fluoro-deoxyglucose (^18^F-FDG) PET coupled with CT (PET/CT) to diagnose certain malignancies led to the incidental (re)-discovery of brown adipose tissue (BAT) in adult humans approximately 15 years ago (9, 10). This finding has re-ignited interest in this approach to treat obesity (11), as BAT activation in rodents increases EE and improves insulin sensitivity (12) and dyslipidaemia (13). Thereafter, a number of elegant studies have been undertaken to determine the role, importance and regulation of BAT in humans. This review will discuss the recent advances in our understanding of the physiology and pathophysiology of human BAT, potential approaches to activate BAT and discuss whether this tissue represents a viable therapeutic target for obesity and its associated metabolic disease.

## The distribution and function of brown and beige adipose tissue

**Figure 2:**
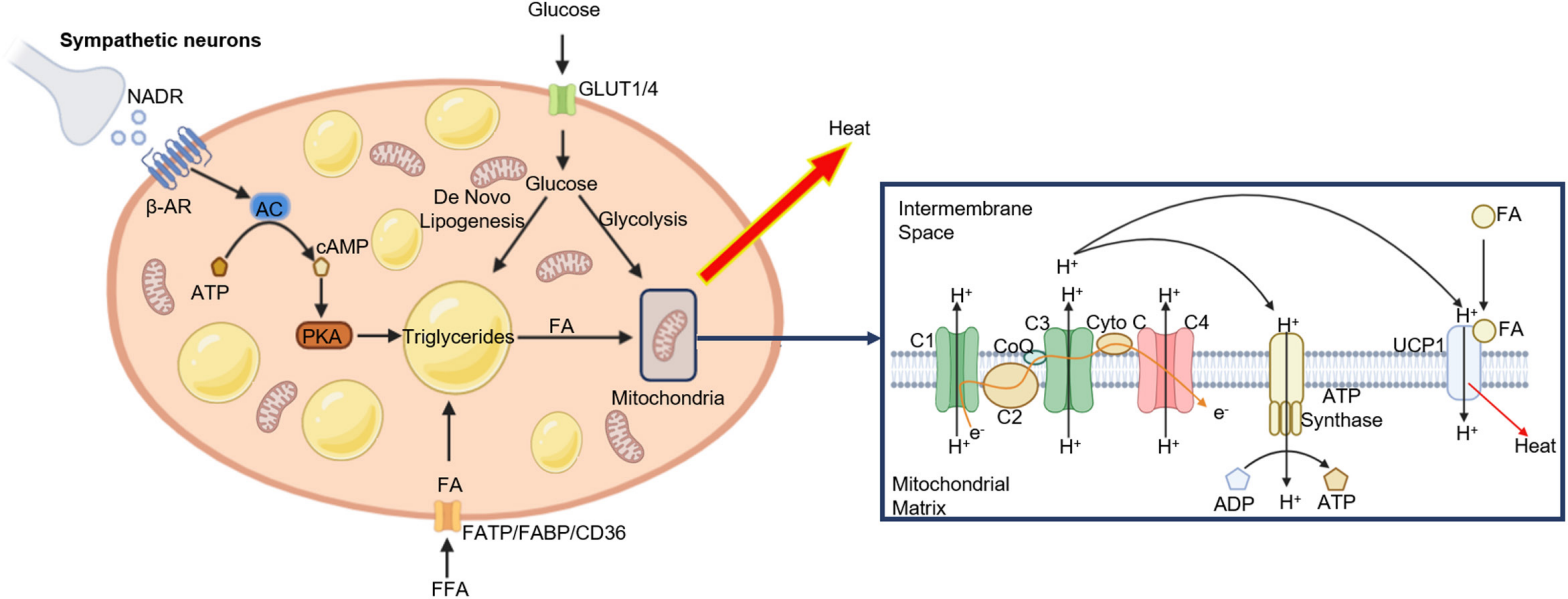
Brown adipocyte activation and molecular mechanism of UCP1 function. Upon cold stimulus, sympathetic neurons innervating BAT release noradrenaline (NADR) from the synapse. NADR binds to various β-adrenergic receptors (β-AR) on the brown adipocyte which activates adenylyl cyclase (AC), converting ATP to cyclic adenosine monophosphate (cAMP). cAMP activates protein kinase A (PKA) which stimulates the lipolysis of triglyceride stores and release of fatty acids (FA). FAs are the primary substrate for thermogenesis but also bind and activate uncoupling protein 1 (UCP1) located in the mitochondria. UCP1 generates heat via transport of protons (H^+^) across the inner mitochondrial member using the electrochemical proton gradient generated by the electron transport train, uncoupling respiration from ATP synthase. Uptake of circulating free fatty acids (FFA) and glucose contribute to the regeneration of intracellular triglyceride stores, additionally glucose can be oxidised and enter the tricarboxylic acid (TCA) cycle. FFAs are transported into the cell by fatty acid transport protein (FATP, fatty acid binding protein (FABP), and cluster of differentiation 36 (CD36). Glucose is transported into the cell via the glucose transporters GLUT1 and 4. C1–4, complex 1–4; CoQ, co-enzyme Q; Cyto C, cytochrome C; e^−^, electron.

Predominantly an organ for energy storage, WAT is widely distributed throughout the body and divided into s.c. and visceral depots. Conversely, the primary function of BAT is to generate heat to maintain the body temperature through non-shivering thermogenesis (NST) during cold exposure (14) and is located in the cervical, supraclavicular, axillary, paraspinal, and perirenal regions (Fig. 1) (15, 16, 17). Unlike WAT, BAT contains multilocular lipid droplets and a high number of mitochondria expressing the thermogenic protein mitochondrial brown fat uncoupling protein 1 (UCP1) (18). When activated, UCP1 dissipates the proton electrochemical gradient across the inner mitochondrial matrix with the energy released as heat in a process termed 'uncoupling' (Fig. 2) (14). Cold exposure stimulates the sympathetic neurones innervating BAT to release noradrenaline which activates β-adrenergic receptors (AR) (classically β3-AR but also β1- and β2-AR (19, 20, 21, 22)). β-AR activation triggers a signalling cascade which results in the hydrolysis of local triglycerides, releasing fatty acids (FA) that activate UCP1 (Fig. 2). In addition to triglyceride stores, BAT sequesters and utilises several circulating substrates such as glucose, fatty acids and some amino acids during thermogenesis (reviewed in (23)). Therefore, BAT activation may improve other metabolic health parameters such as hyperglycaemia and dyslipidaemia in addition to increasing energy expenditure.

In rodents, two distinct types of thermogenic adipose tissue have been identified, classical BAT and beige or brite. Classical BAT is found mainly in the interscapular region and is derived from myogenic factor 5-positive precursors (24), whilst beige fat is found primarily in the inguinal depot and is derived from different progenitor cells (reviewed in (25)). Beige adipocytes are recruited in response to cold or β-adrenergic stimulation (26), express UCP1 (although levels are substantially lower than in BAT) (26) and contribute to thermogenesis (27, 28). Also thermogenic in nature, human BAT has comparable UCP1 function to rodent BAT (18) and interestingly, the molecular signature of human BAT shares similarities with both rodent classical BAT and beige adipose tissue (29).

## The importance of brown adipose tissue in adult humans

Following the discovery of BAT in adults via clinical ^18^F-FDG-PET/CT scans, healthy volunteer studies confirmed that cold exposure substantially increased ^18^F-FDG uptake by BAT (17, 30, 31) and ^18^F-FDG-PET/CT has become the most commonly used technique to quantify the BAT mass and activity (32). During warm conditions, BAT remains metabolically active, with greater glucose and fatty acid uptake compared to that of WAT (33, 34). Clinical ^18^F-FDG-PET/CT scans performed at room temperature demonstrate that ~5–10% of individuals have detectable ^18^F-FDG uptake by BAT (35); however, in dedicated studies when subjects are exposed to cold the prevalence is as high as 95% in young healthy men (17). BAT mass is substantially lower than WAT mass (~16–22 kg) even in normal weight adults (36, 37). The quantity of detectable BAT in humans ranges from ~10–300 g (30, 38, 39, 40), although this may be an underestimate as ^18^F-FDG-PET may not identify all BAT depots and total BAT mass may be as high as ~2550 g (15). However, ^11^C-acetate PET (used to measure BAT oxidative activity) has not revealed novel BAT depots without substantial glucose uptake (41) indicating that ^18^F-FDG-PET/CT estimates may be accurate.

### Regulation and dysregulation of human BAT activity

Clinical PET/CT studies identified that increased outdoor temperature and male sex were associated with reduced ^18^F-FDG uptake by BAT (40, 42). However, dedicated cold exposure studies have not revealed substantial differences in BAT activity between sexes (43), potentially indicating that females activate their BAT at higher room temperature than males. Ethnicity may also alter BAT mass/activity, which has been implicated in the greater risk of metabolic disease in individuals of South Asian origin (44). Perhaps, the most interesting observation was that reduced ^18^F-FDG uptake by BAT was observed with increasing age, fasting glucose and body weight, implicating dysregulation of BAT activity in metabolic disease (17, 31, 42, 45). In addition, obese subjects have reduced fatty acid (using the PET tracer ^18^fluoro-6-thia-heptadecanoic acid) uptake by BAT during both warm and cold exposure in keeping with decreased BAT mass and activity (34), although greater insulin resistance may also contribute to the reduced glucose/fatty acid uptake by BAT in obesity (46). Dysfunctional BAT in obese subjects could reduce EE and contribute to weight gain, as observed in mice with selective disruption of *Ucp1* that develop obesity when housed at thermoneutral conditions (47). However, *Ucp1*^*−*/*−*^ mice housed below thermoneutrality have resistance to diet-induced obesity due to decreased metabolic efficiency, highlighting that dysfunctional BAT does not necessarily cause weight gain and is dependent on the environmental conditions (48). BAT mass and ^18^F-FDG uptake by BAT are also substantially reduced in older subjects (49) and in those with T2DM (49, 50), although interestingly oxidative metabolism is maintained, indicating that functional BAT is preserved in these cohorts (49). These data highlight a critical issue in the therapeutic potential of activating BAT, as the target patient groups require enough BAT mass and function to benefit from activation. Therefore, effective expansion of BAT mass will likely be required to obtain improvements in metabolic health.

There are substantial data that BAT mass can expand or regress in response to different stimuli. For example, in colder climates, greater BAT mass is found in individuals who work outdoors compared with indoor workers (51). Furthermore, repeated intermittent cold exposure for ~7–10 days increased BAT mass and glucose uptake (using ^18^F-FDG-PET/CT), BAT oxidative metabolism, NST and wider cold-induced thermogenesis (CIT) (38, 52). Rare diseases also highlight the plasticity of BAT, as previously mentioned BAT activation is under sympathetic control and patients with catecholamine-secreting tumours (called phaeochromocytomas) often have substantial BAT mass and function which regress upon surgical removal of the tumour (53, 54). Importantly, BAT function can be increased in obese subjects both with and without T2DM (the target patient group) by weight loss, which increased ^18^F-FDG uptake by BAT, BAT volume and non-shivering thermogenesis in some subjects (50, 55, 56). These data suggest that brown adipocyte precursors are present in individuals without detectable BAT and can differentiate into functional brown adipocytes upon appropriate stimulation. Therefore, treatments to increase BAT mass may be successful in patients with metabolic disease. These data also indicate that reduced BAT mass may be a consequence of obesity and it is to be determined whether activating BAT can cause weight loss in obese individuals. However, there are substantial differences in capacity and function of BAT even in healthy individuals, and further research is needed to determine the causes of this variability and whether reduced or absent BAT mass and function can be rescued in all subjects. In addition, it is important to consider other factors regulating BAT mass and function in individuals. For example, BAT activity demonstrates a circadian rhythm in both rodents and humans (57, 58) while dietary composition and timing of feeding/fasting have powerful effects on BAT activity and browning at least in rodents (59, 60, 61). Exercise may also regulate BAT activity, as seen in endurance-trained athletes who have reduced ^18^F-FDG uptake by BAT during cold exposure compared with sedentary adults (62, 63).

### Quantification of cold-induced thermogenesis and energy expenditure by BAT

**Figure 3:**
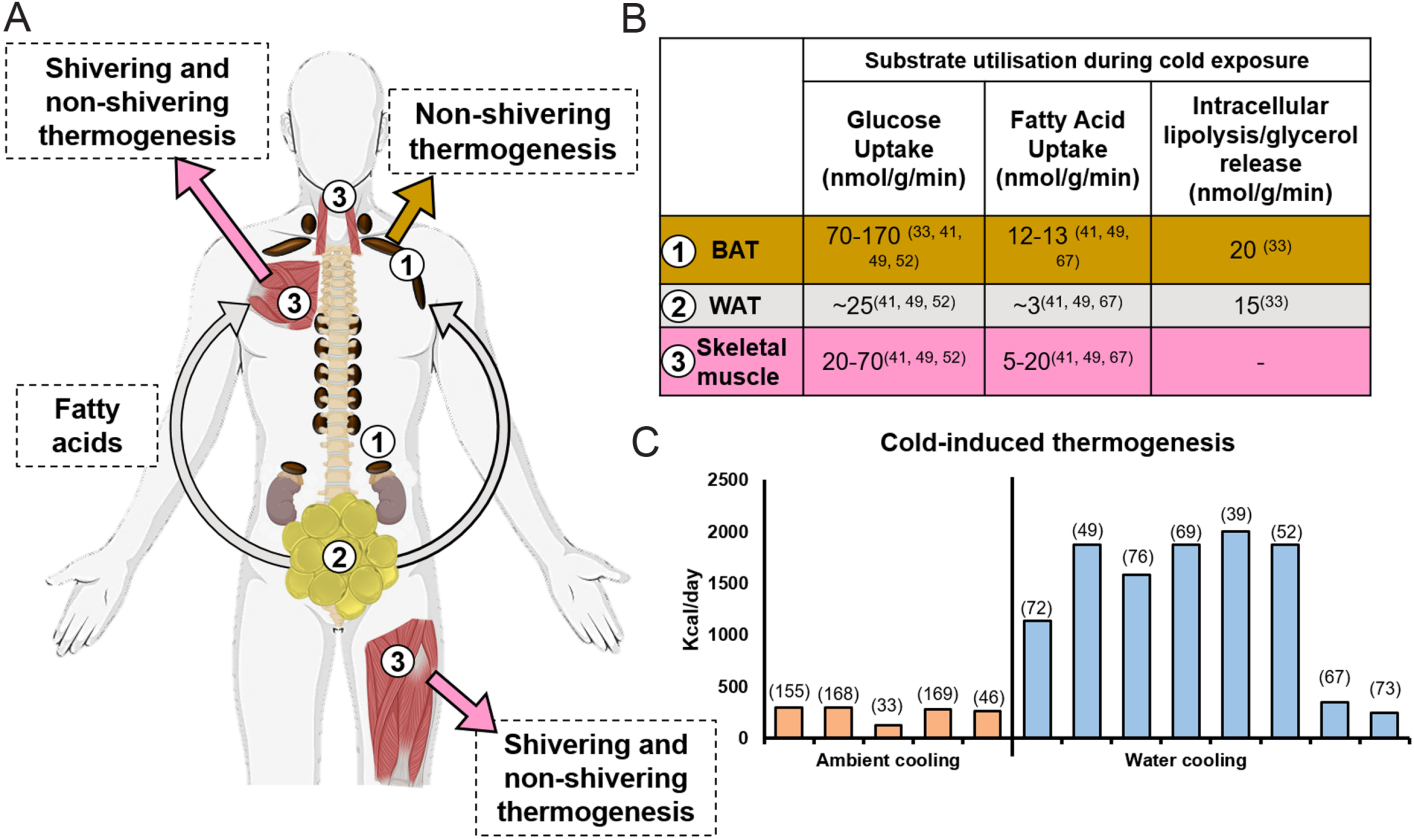
Whole body cold-induced thermogenesis and substrate utilisation. (A) Cold exposure stimulates WAT lipolysis to provide FAs for utilisation by both BAT and skeletal muscle (grey arrows). BAT uses FAs released from intracellular triglyceride stores to fuel non-shivering thermogenesis (orange arrow) but also sequesters circulating FAs and glucose. Skeletal muscle shivering accounts for the largest proportion of whole body heat production, glucose and FA uptake during cold-induced thermogenesis (CIT) (pink arrows). Muscles that contribute substantially to shivering thermogenesis include the longus colli, sternocleidomastoid, pectoralis major, and the rectus femoris. (B) During cold exposure, glucose uptake per gram of tissue is greater in BAT than skeletal muscle but with similar fatty acid uptake. However, whole body FA and glucose uptake by BAT is comparatively low due to substantially greater skeletal muscle mass. (C) Quantification of CIT varies greatly depending on the cooling method used and temperature, ambient air cooling protocols (orange columns) typically elicit a lower increase in energy expenditure compared to water cooling blanket/suit protocols (blue columns), but substantial CIT is induced by both methods. Additional references used for data in panel C (168,169).

Determining the maximal capacity of BAT is key to understanding its therapeutic potential. Early research estimated that 50 g of activated human BAT could increase EE by 20% above basal metabolic rate (64). In addition, EE increases by ~250–300 kcal/24 h during mild cold exposure (Fig. 3) and CIT is higher in subjects with greater BAT mass in some (65, 66) but not in all studies (67). However, the use of ^15^O_2_-PET suggested that BAT only accounts for a very small contribution to CIT, <20 kcal/24 h even in subjects with substantial BAT mass (67, 68). Thus, unlike in rodents, non-shivering thermogenesis accounts for a small proportion (~1%) of CIT in humans, indicating approaches to activate BAT alone will not significantly increase whole body EE. However, it is interesting to note that the deep muscles (particularly in the neck) located adjacent to BAT are responsible for the majority of CIT (67, 69). It is possible that greater sympathetic activation in subjects with BAT also increases skeletal muscle thermogenesis, or that BAT secretes factors that enhance EE in skeletal muscle in a paracrine fashion, as seen in rodents (70). In addition, interventions that increase BAT mass also increase wider CIT, highlighting the potential benefits of this approach (38). For example, repeated cold exposure at 17°C for 2 h/day for 6 weeks increased cold-induced ^18^F-FDG uptake by BAT, CIT by ~200 kcal/day and reduced fat mass (66). This substantial increase in EE during acute cold exposure highlights the potential benefits of activating this pathway, to put this into context increasing EE by 50–60 kcal/day for 1 year would result in weight loss of ~2.5 kg (71) if there was no compensatory increase in food intake. Further research to dissect the pathways regulating CIT may identify novel targets for manipulation to increase EE.

### Metabolic effects of BAT activation and cold-induced thermogenesis

The metabolic benefits of BAT activation and wider CIT extend beyond burning calories. During warm exposure when thermogenesis is not required, BAT sequesters and utilises circulating metabolic substrates such as glucose and FAs to a greater extent than WAT (33, 34). However, uptake of these substrates and others such as glutamate by BAT increases substantially following cold activation, in addition to hydrolysis and oxidation of its own triglyceride stores (33, 72). CIT also leads to increased glucose and FA uptake by skeletal muscle and lipolysis in WAT (41, 49, 52, 67, 73). Highlighting the substantial metabolic activity of BAT, glucose and potentially FA uptake are greater per gram of tissue in BAT than in either skeletal muscle or WAT (Fig. 3). However, skeletal muscle mass (~30 kg) is substantially greater than BAT mass and accounts for ~50% of whole body glucose uptake during cold, compared with ~1% for BAT (72).

As mentioned previously, in line with increased substrate utilisation BAT activation improves glucose homeostasis. For example, acute cold exposure in overweight/obese men increased glucose disposal and oxidation in addition to insulin sensitivity in subjects with detectable BAT, these changes were not observed in the ‘BAT negative’ group (74). Furthermore, 4 weeks of mild cold exposure (19°C for 10 h/night) in lean healthy men improved postprandial insulin sensitivity, reduced leptin levels and increased adiponectin concentrations in addition to increasing ^18^F-FDG uptake by BAT (39). In T2DM subjects, cold exposure (~15°C for 2–6 h/day for 10 days) improved whole body insulin sensitivity (primarily through increased glucose disposal) and increased ^18^F-FDG uptake by BAT and skeletal muscle but not WAT (50).

BAT activation is also associated with changes in circulating lipids. For example, in overweight/obese men those with greater BAT activation had increased cold-induced lipolysis, FA oxidation and adipose tissue insulin sensitivity (75). In addition, acute cold exposure decreased triglyceride and very low-density lipoproteins (VLDL)-cholesterol concentrations in those subjects the following day, suggesting BAT activation may have prolonged beneficial effects. This may be in part due to sequestration of fatty acids derived from triglyceride-rich lipoproteins (TRLs) (76). In addition, BAT thermogenesis is activated to a similar extent by a meal as by cold, this postprandial thermogenesis utilises TRL-derived FAs and glucose which may improve systemic FA oxidation in addition to glucose disposal (73). These data suggest that BAT activation and wider CIT may improve insulin resistance and dyslipidaemia in addition to increasing EE, making activation of this pathway an attractive prospect to treat metabolic disease. BAT activation may also have additional beneficial effects, for example short-term cold exposure reduced local inflammation within fat depots (77) and the wider benefits of BAT activation and CIT remain relatively unexplored.

## Approaches to activating BAT

As described above, there are clear metabolic benefits from acute activation of BAT and wider CIT, questions remain as to whether these improvements will be maintained during chronic activation. In addition, a major challenge for the field is how to safely achieve long-term expansion and activation of BAT.

### Cold exposure

To date, the most common method to activate BAT in humans is either to reduce ambient room temperature to ~16–19°C (30, 33) or use a cold water-infused suit/jacket (41, 49). Both methods elicit similar levels of BAT activation at least as measured by glucose uptake (33, 41, 46, 52). Repeated cold exposure for several hours per day for up to 6 weeks increased BAT mass/activity (as measured using ^18^F-FDG), CIT and decreased fat mass (38, 50, 66). Although it is possible that the increased ^18^F-FDG uptake demonstrates the activation of previously dormant BAT, in rodents repeated cold exposure leads to differentiation of new thermogenic beige adipocytes (78) and the same is most likely true in humans. Importantly, these studies reveal that short-term cold exposure improves cardiometabolic markers and potentially decreases fat mass in humans without the need for pharmacotherapy. However, this technique is time-consuming and may be uncomfortable for patients. It is also unclear whether these benefits are maintained over time and ongoing studies will determine whether chronic cold exposure (or repeated short-term cold exposure for several months) improves metabolic health (79, 80).

Increased ambient temperature is associated with the prevalence of obesity in some (81, 82) but not in all studies (83). Indoor housing temperatures in the UK have increased since the 1970s, potentially due to greater use of central heating including in more energy-efficient homes (84, 85, 86). The reduced requirement for CIT due to warmer ambient temperatures could lower EE (Fig. 3) and contribute to the increased prevalence of obesity. Therefore, a concerted effort to reduce room temperature through the reduced usage of central heating to increase EE may have metabolic benefits in addition to being the most cost-effective ‘therapeutic’ option in cold climates such as the UK. However, in rodents, intermittent cold exposure causes a compensatory increase in food intake to meet the increased thermogenic demands (12), while in humans increased EE during cold exposure results in a parallel increase in*ad libitum* food intake (87). Therefore, while cold exposure may improve metabolic health this may not necessarily result in weight loss.

### Pharmacotherapy for BAT activation

Pharmacotherapy to activate BAT (and potentially other tissues involved in CIT) is an attractive option as this would be a more comfortable method of activation. However, a subject must have enough BAT to respond to a ‘BAT activator’ particularly as the target patient group (typically obese subjects with T2DM who may be older) generally have very little BAT. Therefore, the ideal drug would expand BAT mass in addition to activating BAT. As BAT expansion and activation are both under sympathetic regulation (53, 88), the majority of research in this area has focused on the effect of sympathetic agonists.

#### Sympathomimetics

Activation of the β3-AR induces browning and BAT thermogenesis, while administration of β3-agonists induces weight loss and improves hyperglycaemia in rodents (89). Consequently, there was significant interest in β3-AR agonists in humans even prior to the recent identification of BAT in adult humans (90, 91). Β3-agonist administration for 4–8 weeks in humans improved lipids and insulin sensitivity although there was no effect on body weight (92, 93). More recently, a single high dose (200 mg) of the β3-AR agonist mirabegron (licensed for urinary frequency/incontinence) in humans housed at 23°C increased ^18^F-FDG uptake by BAT and increased EE by ~200 kcal/24 h (19). In addition, an administration of 100 mg mirabegron daily for 4 weeks in healthy women increased BAT mass and volume (using ^18^F-FDG) and increased EE but did not alter body weight (94). In accordance with the earlier studies, mirabegron improved insulin sensitivity and increased high-density lipoprotein cholesterol in these subjects. However, a lower dose of mirabegron (50 mg) did not activate BAT thermogenesis (22) and the effects at higher doses may be due to off target activation of particularly the β2-AR and also β1-AR that are more highly expressed in human BAT than β3-AR (21, 22). These data highlight the difficulties with developing selective adrenergic receptor agonists to activate BAT.

The mixed adrenoreceptor agonist ephedrine (which also inhibits noradrenaline re-uptake in post-synaptic neurons (95, 96)) also increased ^18^F-FDG uptake by BAT in lean (but not obese) adults at room temperature (97). However, ephedrine also increased heart rate and blood pressure, side effects also induced by mirabegron that limit the potential of this approach (19, 94, 97). In addition, lower dose ephedrine (1 mg/kg) did not acutely activate BAT in healthy humans (98) while chronic administration may in fact reduce BAT activity (99). These data suggest that chronic sympathetic stimulation may result in desensitisation in BAT.

#### Thyroid hormones

Thyroid hormone receptor activation is crucial for BAT thermogenesis and adrenergic responsiveness in mice (100). Similarly, individuals with thyrotoxicosis have increased BAT glucose uptake, lipid oxidation, EE, and possibly improved insulin sensitivity; these changes are reversed once euthyroidism is restored (101, 102). A recent trial also demonstrated that administration of thyrotropin-releasing hormone in healthy subjects increased glucose uptake by BAT but only during cold exposure (103). While these studies highlight the importance of the thyroid in BAT activation, the long-term effects of thyroid hormone administration on BAT function are unknown.

#### Other drugs known to activate BAT

Capsaicin and capsinoids are substances naturally present in chilli peppers that are agonists of the transient receptor potential vanilloid type 1 (TRPV1) receptor (104). In rodents, capsinoids stimulate sympathetic activation of BAT and increase UCP1 expression in both BAT and WAT (104, 105). In healthy humans, acute ingestion of capsinoids (9 mg) significantly increased whole body EE only in subjects with detectable BAT (106). Chronic capsinoid supplementation (9 mg daily for 6 weeks) increased CIT in healthy subjects (66) and potentially increased resting EE in overweight individuals (107), although whether these effects are mediated by BAT is unclear. These data provide proof-of-concept that dietary supplementation could be a relatively safe method to increase EE and BAT activity.

Produced by the liver and modified by gut microbiota, bile acids (BA) are released into the intestinal lumen and circulation in the postprandial period and regulate metabolism (reviewed in (108)). In rodents, BAs increase BAT thermogenesis and induce browning of WAT through the G-protein-coupled bile acid receptor TGR5 and the cyclic-AMP-dependent thyroid hormone activating enzyme type 2 iodothyronine deiodinase (109, 110, 111). In humans, administration of the BA chenodeoxycholic acid (CDCA) for 2 days increased BAT activation and whole body EE *in vivo* (112) and CDCA increased mitochondrial uncoupling in human brown (but not white) adipocytes through TGR5 (112).

In rodents, acute and chronic glucocorticoid excess decreases UCP1 expression and reduces BAT thermogenesis, conversely in humans acute glucocorticoid excess increases UCP1 and oxygen consumption *in vitro* and increases ^18^F-FDG uptake and heat production by BAT and CIT *in vivo* (113, 114, 115). However, chronic glucocorticoid excess reduces BAT function so would not be an appropriate therapeutic agent (114, 116), but importantly, these data highlight the species-specific regulation of BAT activation.

## Browning agents to enhance thermogenesis

**Figure 4:**
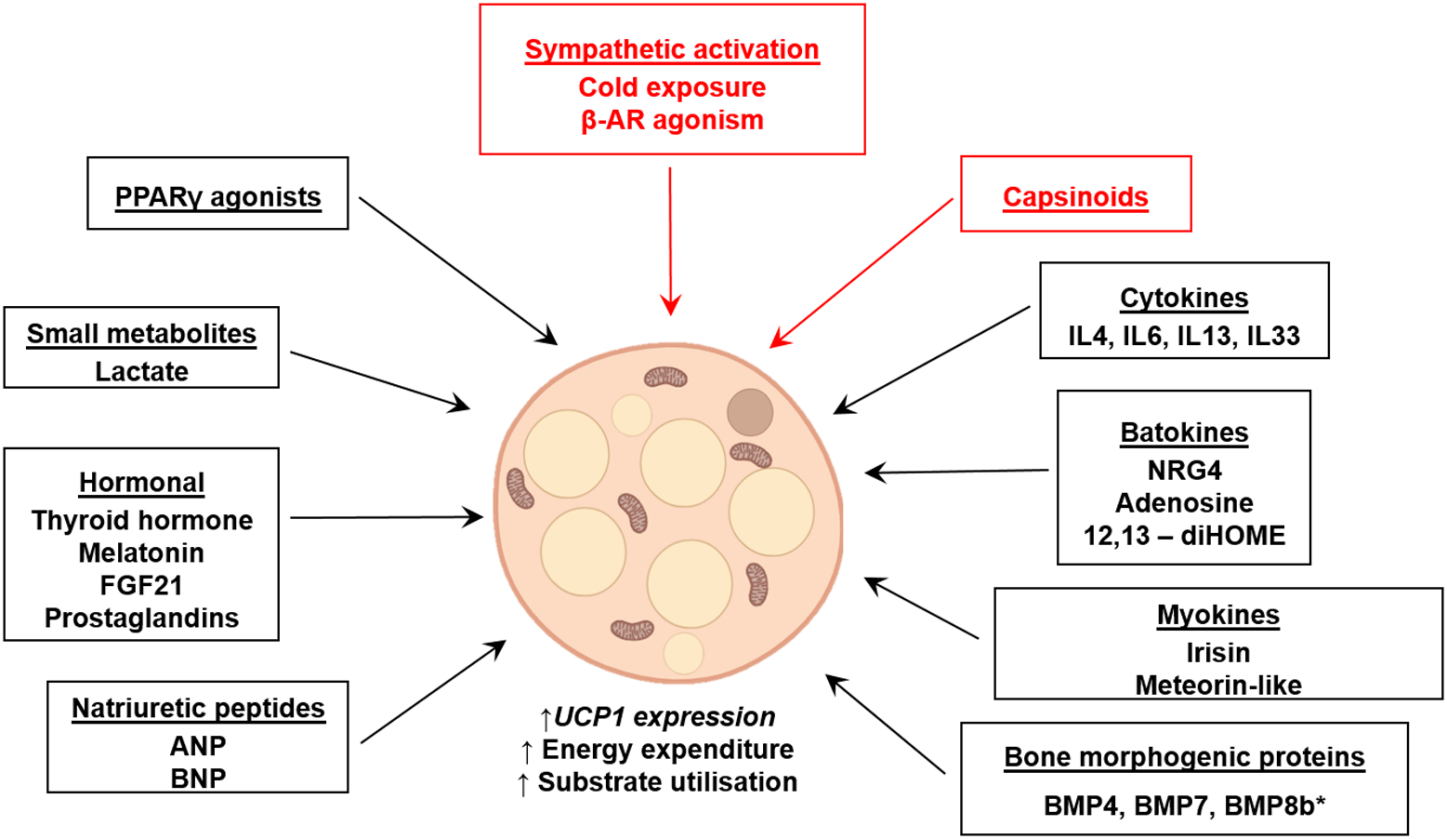
Factors that induce browning of typical WAT depots. An illustration of factors that induce thermogenic beige adipocyte formation* in vivo* and* in vitro* with greater UCP1 expression and uncoupled respiration compared to white adipocytes. The small number of factors that induce browning of white adipose tissue* in vivo* in humans are highlighted in red and underlined. *BMP8b is classed additionally as a BATokine. 12,13-diHOME, 12,13-dihydroxy-9Z-octadecenoic acid; β-AR, β-adrenoreceptor; BMP, bone morphogenic protein; BNP, brain natriuretic peptide; FGF21, fibroblast growth factor-21; IL, interleukin; NRG4, neuregulin-4; PPARγ, peroxisome proliferator-activated receptor-γ.

Due to the relatively small quantity of BAT in adult humans, upon activation, the systemic clearance of glucose and caloric capacity is substantially lower than skeletal muscle (69, 72). Therefore, therapeutic strategy is to expand the thermogenic adipose tissue mass in a process termed 'browning' (Fig. 4). Increasing WAT thermogenesis could have profound metabolic effects as, in obese subjects, WAT accounts for over one-quarter of total body weight (117). Cold exposure is a powerful inducer of browning in rodents (78) and increases supraclavicular BAT mass in humans; however, studies have not demonstrated increased glucose uptake (118) or oxidative capacity (52) (measured by ^11^C-acetate) in classical WAT depots *in vivo* following repeated cold exposure. These data suggest either that repeated cold exposure is not a sufficient stimulus to increase WAT thermogenesis or that classical WAT depots have a low browning capacity. Pharmacotherapy may hold greater promise, browning agents such as β3-agonists can induce formation of UCP1-positive ‘beige’ thermogenic adipocytes in WAT depots in both rodents and humans, although as discussed above their adverse effects limit their potential (26, 119, 120).

Numerous browning agents have been identified in rodents, some of which have been investigated in humans. For example, fibroblast growth factor-21 (FGF21) release is induced by cold and increases UCP1expression in murine WAT and BAT, and in human adipocytes (121, 122, 123). FGF21 is expressed in BAT (123, 124), although hepatic FGF21 primarily accounts for circulating levels and the beneficial metabolic effects (125). FGF21 analogue administration to obese humans with T2DM improved circulating lipids and reduced body weight (potentially by reducing food intake although this was not measured) ,although it did not improve glucose levels (126, 127). However, BAT activity and WAT browning were not measured in these studies so it is unclear if adipose thermogenesis contributed to the observed metabolic improvements, further research is needed to determine if FGF21 administration *in vivo* induces browning in humans. FGF21 also induces bone loss, which lessens the therapeutic potential of this approach (128).

Several bone morphogenic proteins (BMPs, members of the transforming growth factor superfamily) drive brown adipogenesis, notably BMP7. Transgenic disruption of *Bmp7* in mice substantially reduced UCP1expression and BAT mass, while its overexpression increased UCP1 and EE and reduced body mass (129). In addition, BMP7 (and BMP4) induces browning in human adipocyte cell models (130, 131). However, BMP7 may only work as a browning agent below thermoneutrality, which is an important consideration for any therapeutic agent (132). The effect of BMP7 on metabolic health has not been studied *in vivo* in humans, although BMP7 has been FDA approved for bone fracture treatment in clinical trials (133).

Thiazolidinediones are peroxisome proliferator-activated receptor-γ (PPARγ) agonists used as insulin-sensitising drugs for the treatment of T2DM, although adverse side effects have reduced their use substantially (134). In rodents, PPARγ-agonists are powerful browning agents, both *in vivo* and *in vitro* (135, 136). In humans, PPARγ-agonists also induce browning in adipocytes but *in vivo* in fact reduce cold-induced ^18^F-FDG uptake by BAT (137). Another anti-diabetic drug, the dipeptidyl peptidase-4 (DPP-IV) inhibitor sitagliptin decreased body weight, increased energy expenditure and increased UCP1proteinexpression in BAT in obese mice (138). However, DPP-IV inhibition using sitagliptin in overweight pre-diabetic subjects for 12 weeks increased ^18^F-FDG uptake slightly by subcutaneous WAT but not by BAT during cold exposure (139). Therefore, it is unlikely that DPP-IV inhibitors induce substantial browning in humans.

Irisin is another browning agent that has received substantial attention. Irisin is secreted from skeletal muscle during exercise and substantially increases UCP1expression in inguinal WAT, increases EE and protects against weight gain in mice (140). In humans, irisin induced browning in white adipocytes *in vitro* while circulating irisin concentrations are increased by exercise and cold exposure (122, 140). However, irisin had no effect on brown adipocytes and may also induce osteogenesis (141). Data in humans on the effects of exercise programmes on circulating irisin levels and induction of browning are inconsistent, calling into question whether irisin mediates any of the beneficial effects of exercise, although methodologies often vary widely between studies (142, 143). Despite differences in methodology to quantify irisin concentrations, systemic irisin levels are increased in obesity which may reduce the potential of using irisin as a therapeutic agent, although levels are likely decreased in T2DM (144). The effect of irisin administration *in vivo* in humans is yet to be tested. While numerous factors have been identified as browning agents in rodents (145), the above data highlight the importance of assessing the effect of browning agents *in vivo* in humans and at present there are very limited data on the majority of these factors in humans. Β-agonists have successfully demonstrated proof-of-concept but further work is necessary to dissect the mechanisms regulating adipose tissue browning in humans and the thermogenic capacity of various WAT depots to determine the therapeutic potential of this approach.

## Secreted factors from BAT

As previously discussed, the presence of BAT is associated with favourable metabolic profiles (74) and there is recent evidence BAT secretes factors (often termed 'BATokines', although most if not all of these factors are also secreted from other tissues) with beneficial paracrine and endocrine functions. Consequently, there is substantial interest in identifying BATokines with therapeutic potential. Many factors have been identified, the vast majority in rodents. BMP8b has gained interest as a BATokine as its expression is induced in BAT by cold exposure and BMP8b directly enhances sympathetic-stimulated BAT thermogenesis (146). In addition, secreted BMP8b increased adipose tissue browning through enhanced sympathetic innervation and vascularisation even at thermoneutrality in mice through secretion of another BATokine neuregulin-4 (NRG4) (147). NRG4 also exerts beneficial metabolic effects in other tissues such as the liver where it inhibits *de novo* lipogenesis (148) and increases FA oxidation, while NRG4 also increases glucose uptake in skeletal muscle (149). In humans, NRG4 expression in WAT is reduced in obese subjects and circulating levels are lower in non-alcoholic fatty liver disease (150). However, the contribution of BAT to total serum NRG4 is unknown and therapeutic administration has not been tested in humans.

In both rodents and humans, circulating concentrations of the BATokine 12,13-dihydroxy-9Z-octadecenoic acid (12,13-diHOME) are increased following cold exposure (151) and exercise (152). In mice, 12,13-diHOME administration increased fatty acid uptake by brown/beige adipocytes and skeletal muscle (151, 152). In addition, plasma 12,13-diHOME concentrations in humans were inversely associated with fat mass, fasting insulin and triglyceride concentrations (153); however, the effect of 12,13-diHOME administration to humans is currently unknown. Adenosine is an another BATokine that activates BAT and induces browning *in vivo* in mice and *in vitro* in humans, via activation of the A_2A_ receptor (154). Intravenous adenosine administration in lean healthy men *in vivo* increased BAT but not WAT perfusion in keeping with increased BAT oxidative metabolism (155). Further work is required to determine whether prolonged activation of A_2A_ receptors induces browning in humans *in vivo*. Recent work has focused on identifying BATokines in humans. For example, comparative analysis of the human brown and white adipocyte secretome recently identified ~100 human BATokines (156), and through this ependymin-related protein 1 was identified as an important regulator of both thermogenic differentiation and noradrenaline-stimulated thermogenesis in human brown adipocytes (156). Further work investigating the role of the BAT secretome in humans is needed to determine if any BATokines offer a therapeutic potential.

## Potential concerns with BAT activation as a therapeutic strategy

The metabolic benefits of BAT activation and cold exposure make increasing EE an attractive target for obesity and metabolic disease; however, there are concerns with this approach that must be taken into consideration. For example, selective activation of BAT may prove difficult to achieve or even be desired, as evidenced by selective β3-AR agonists that may in fact require activation of other β-receptor subtypes in BAT, and part of their beneficial effects may be mediated by other tissues (19, 21, 22, 94). Chronic sympathetic activation (e.g. from β3-AR agonists (19, 94), thyrotoxicosis (101) or from supraphysiological thyroid hormone replacement (157)) causing tachycardia and hypertension may result in unacceptable cardiovascular side effects such as myocardial infarction or stroke (158). It is also possible that elevated heart rate is essential for increased EE and additional research is required to identify whether there is a safe threshold of heart rate that does not increase cardiovascular risk (159). Further research to identify pharmacological mechanisms to safely activate BAT and/or wider cold-induced thermogenesis is urgently required.

Another potential issue is dissipation of the heat generated by pharmacological BAT activation. BAT activation prevents reduced body temperature during cold exposure, however, when activated chronically at room temperature or above thermoneutrality there is a risk of inducing hyperthermia. For example, 2,4-Dinitrophenol (DNP) was used as a weight loss medication as early as the 1930s (160). DNP caused generalised uncoupling of oxidative phosphorylation, leading to hyperthermia particularly during overdose that could be fatal (161, 162). Whilst selective BAT activation is unlikely to cause such thermal stress due to its low quantity, any pharmacological approach to activate BAT will have to be specific and avoid off target effects.

Finally, an approach to increase EE may cause a compensatory hyperphagic response, as observed in mice and potentially humans during cold exposure, to meet the increased metabolic demands (12, 87, 163). However, not all pharmacotherapy that activates BAT in rodents causes hyperphagia (164), so it is unclear whether weight loss from increased EE by BAT may be neutralised by increased caloric consumption. Synergistic combination therapy with appetite suppressants may be required to maintain the benefits of BAT activation (165).

## Perspective and conclusion

Since the identification of BAT in adult humans, there has been a resurgence in investigation of BAT activation and wider thermogenesis as a therapeutic strategy for obesity and metabolic disease. Thanks largely to PET imaging and to other novel *in vivo* techniques for measuring human BAT activity, significant progress has been made in understanding the role and regulation of human BAT, although to date most of the metabolic benefits from BAT activation have occurred in the context of acute cold exposure. In addition, pharmacological activation of BAT has been demonstrated at room temperature in important proof-of-concept studies but more research is required to fully understand the pathways regulating adipose tissue thermogenesis in order to develop treatments to safely activate BAT. Recent innovative approaches in rodents have identified the therapeutic potential of increasing BAT mass to treat metabolic disease, as evidenced by transplantation of either BAT (166) or beige adipocytes (167) which improves weight loss, glucose homeostasis and insulin sensitivity. While this approach remains untested in humans, it provides a clear proof of principle that increasing BAT mass improves metabolic health, which justifies further research to increase BAT mass and activity in humans. Pharmacological browning of white adipose tissue offers a larger adipose depot to increase EE and act as a glucose and lipid sink, although it remains unknown how much browning of these depots is possible. These current data suggest that selective BAT activation without significant expansion of BAT mass would not increase EE sufficiently to induce weight loss in humans and the most effective use of BAT activators may be as treatments for the comorbidities associated with obesity such as hyperglycaemia and dyslipidaemia rather than obesity itself. Further research into chronic BAT activation, potentially in combination with other approved weight loss therapies such as appetite suppressants, will determine whether BAT activation can complement current treatment options.

## Declaration of interest

The authors declare that there is no conflict of interest that could be perceived as prejudicing the impartiality of this review.

## Funding

This work was supported by the Medical Research Council (MR/K010271/1, MR/S035761/1), the Chief Scientist Office (SCAF/17/02), and the British Heart Foundation.

## Author contribution statement

B T M and R H S wrote the manuscript and K J S critically revised the manuscript. All authors approved the submitted version.
